# A bibliometric analysis of the current state of research on family interventions for ASD

**DOI:** 10.3389/fpsyt.2025.1435612

**Published:** 2025-05-08

**Authors:** Xuhui Meng, Xue Zhou, Jiahui Luo, Jiaxia Li, Li Zhou, Yuhan Zhang

**Affiliations:** ^1^ School of Rehabilitation Medicine, Jiamusi University, Jiamusi, China; ^2^ The Third Affiliated Hospital of Jiamusi University, Jiamusi University, Jiamusi, China; ^3^ Heilongjiang Provincial Key Laboratory of Children's Neurological Rehabilitation, Jiamusi, China; ^4^ Heilongjiang Children Cerebral Palsy Prevention, Treatment and Education Center, Jiamusi, China

**Keywords:** autism spectrum disorders, family interventions, bibliometrics, CiteSpace, early intervention

## Abstract

In recent years, family intervention has become a hot research direction in the field of autism spectrum disorder (ASD) treatment. A large amount of research literature shows that family intervention is effective in ASD rehabilitation. Still, the concept of family intervention is vaguely defined, and there is a lack of descriptions of specific intervention programs and treatment pathways and a diversity of intervention programs have been reported. In this study, we conducted an econometric analysis of the literature on family intervention for ASD to clarify the concept of family intervention and to analyze and explore the research hotspots, effective family intervention models for ASD, intervention techniques, and possible future research directions in this field.

**Methods:**

A metrological analysis of the literature related to ASD family interventions on the Web of Science from 1987 to 2024 was conducted using CiteSpace software to map the network of countries/institutions, journals, authors, co-cited literature, and keywords in the field. The results were visualized and analyzed.

**Results:**

A total of 1,891 documents were retrieved. The United States (1,028) led in the number and relevance of publications, followed by Canada (254) and Australia (209). The trend of publications was upward. Baranek was the most published author (19) and the University of California System was the most published university. Developed countries dominate the research. According to the timeline graph, it can be learned that the current research hotspots in this field are mostly focused on early intervention in family-based, psychological stress in parents of children with autism.

**Conclusion:**

This visual analysis identifies the most influential institutions and countries, as well as cited journals and authors in the field of family therapy autism research. The direction of research in family therapy for autism should be to find effective treatments for autism based on the home environment, and currently, the most prominent family therapy for autism is telemedicine and parent-mediated intervention. The future direction of this research area could be taken by artificial intelligence techniques specifically applied to children with autism in a computer context.

## 1 Introduction

Autism spectrum disorder (ASD) is a common neurodevelopmental disorder characterized by impairments in social interaction, communication deficits, restricted interests, and repetitive behaviors (1, 2). The Centers for Disease Control and Prevention (CDC) reported a 2.8% prevalence of ASD in 2023, translating to approximately 1 in 36 individuals globally (3). While understanding of ASD has advanced, its etiology remains multifactorial, involving genetic, neurobiological, and environmental factors (4, 5). Despite progress in intervention research, long-term outcomes for individuals with ASD remain challenging. Most individuals with co-occurring intellectual disabilities achieve daily living independence but require ongoing family support (6). Only a subset of those with cognitive abilities within typical ranges attain independent living (7). Moreover, social outcomes such as marriage and friendships remain limited (8). In recent years, individuals with ASD have increasingly accessed higher education. However, despite cognitive abilities within typical ranges, students with ASD often face academic challenges and high dropout rates (9). Employment outcomes also lag behind other disability groups, with individuals with ASD experiencing significantly lower workforce participation (10). Evidence suggests that intensive vocational training during secondary school and post-school placement programs can strengthen employment pathways for this population (11). Collectively, these findings highlight persistent barriers to societal integration for individuals with ASD.

Family interventions aim to enable caregivers to deliver habilitation in home settings, fostering improved family communication, enhanced wellbeing, and reduced caregiver stress (12–15). Caregivers frequently employ videos, games, and daily communication activities to support skill development in children, including activities of daily living, social engagement, and gradual community integration (16). Recent literature underscores the utility of habilitative approaches such as video-based interventions (17). Notable models include the Early Start Denver Model (ESDM), a naturalistic developmental–behavioral intervention (18), and telemedicine-based strategies (19). However, the clinical efficacy of family-based interventions remains context-dependent, with uncertainty regarding their optimal integration with other habilitative services. Thus, multidisciplinary collaboration is critical to designing evidence-based family intervention strategies (20).

Bibliometric analysis offers a systematic framework to evaluate research trends and guide future efforts (21). CiteSpace facilitates the visualization of knowledge domains, enabling researchers to identify emerging themes and compare methodological approaches (22).

## 2 Research methodology

**Figure 1:**
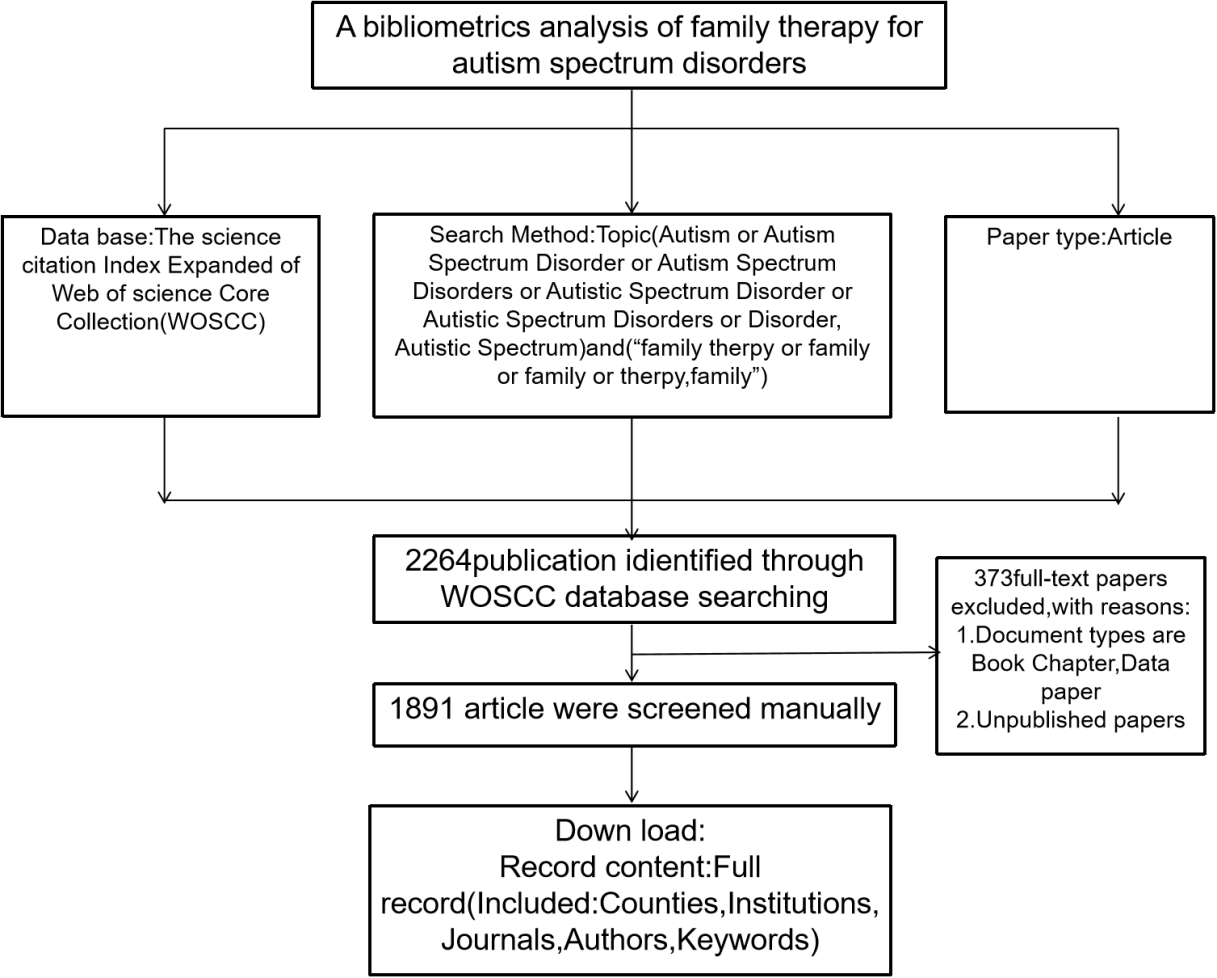
Flowchart of bibliometrics.

The bibliometric research process is illustrated in **Figure 1**. Subject searches were conducted in the Web of Science (WoS) core title database. The search was conducted in the WoS core title catalog. The search was carried out from the date of creation until 1 March 2024. Articles and reviews were the literature types included. The following search terms were entered in the Subject field: (Autism Spectrum Disorder or Autism Spectrum Disorders or Autistic Spectrum Disorder or Disorder, Autistic Spectrum) Autistic Spectrum) and (“family therapy or therapy, family or family intervention” or “family intervention “), language = English. Newspapers, conferences, adverts, and literature containing incomplete and duplicate information were excluded. In the end, a total of 2,201 papers were retrieved. After manual screening, a total of 1,891 articles were included and data were extracted for variables such as title, year of publication, country, institution, journal, author, and keywords. CiteSpace (version 6.1.R3) was used in this study for bibliometric analysis and data visualization. Literature data were downloaded in plain text format, including title, author, abstract, source publication, cited literature, and keywords. Keyword network co-occurrence maps were generated; the data were then imported into CiteSpace 6.1

## 3 Research results

**Figure 2:**
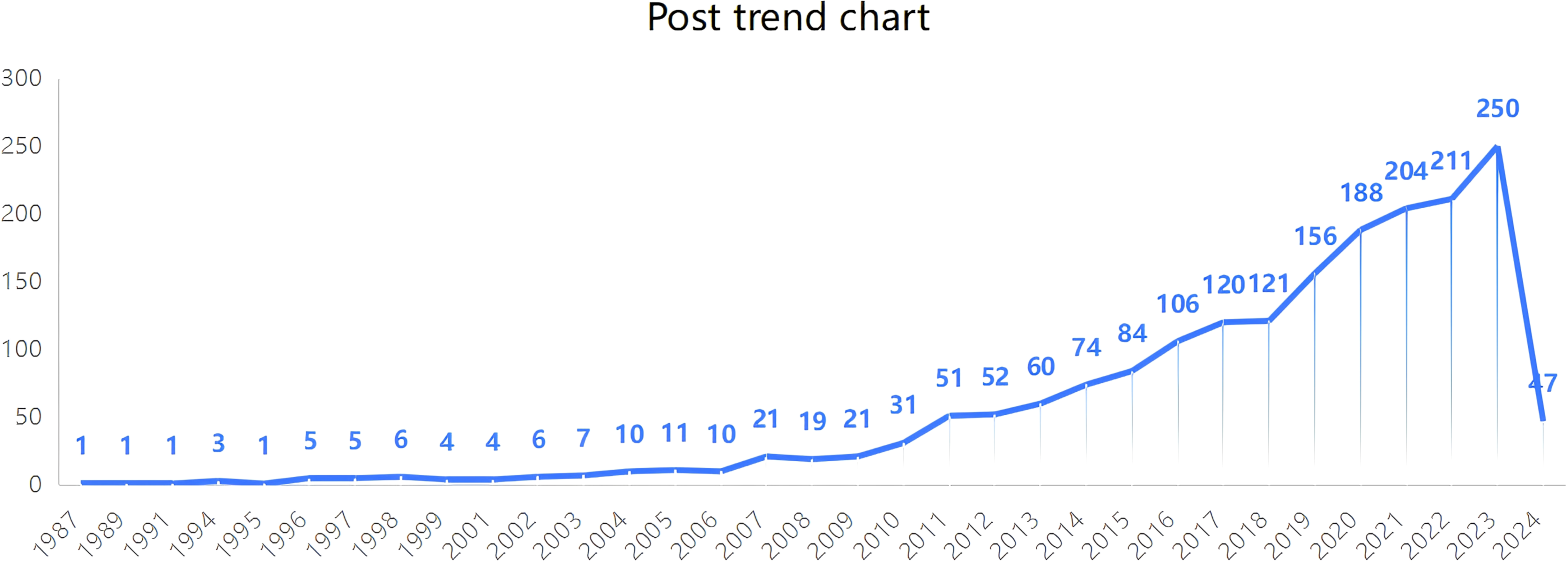
Number of annual publications related to family therapy for autism.

The publication trend search yielded a total of 1,891 relevant articles (see **Figure 1**). The number of articles published from 1987 to 2010 was small and did not change much. During this period, family therapy for autism treatment was still in its infancy. The number of literature in this field started to increase rapidly in 2011 and the development rate increased significantly after 2017. The number of articles in this field peaked in 2023. The number of articles published in 2024 was low, as the year 2024 had not yet passed. The trend shows that family therapy has great potential for the development of this area of autism treatment (see **Figure 2**).

### 3.1 Countries/areas and institutions

**Figure 3:**
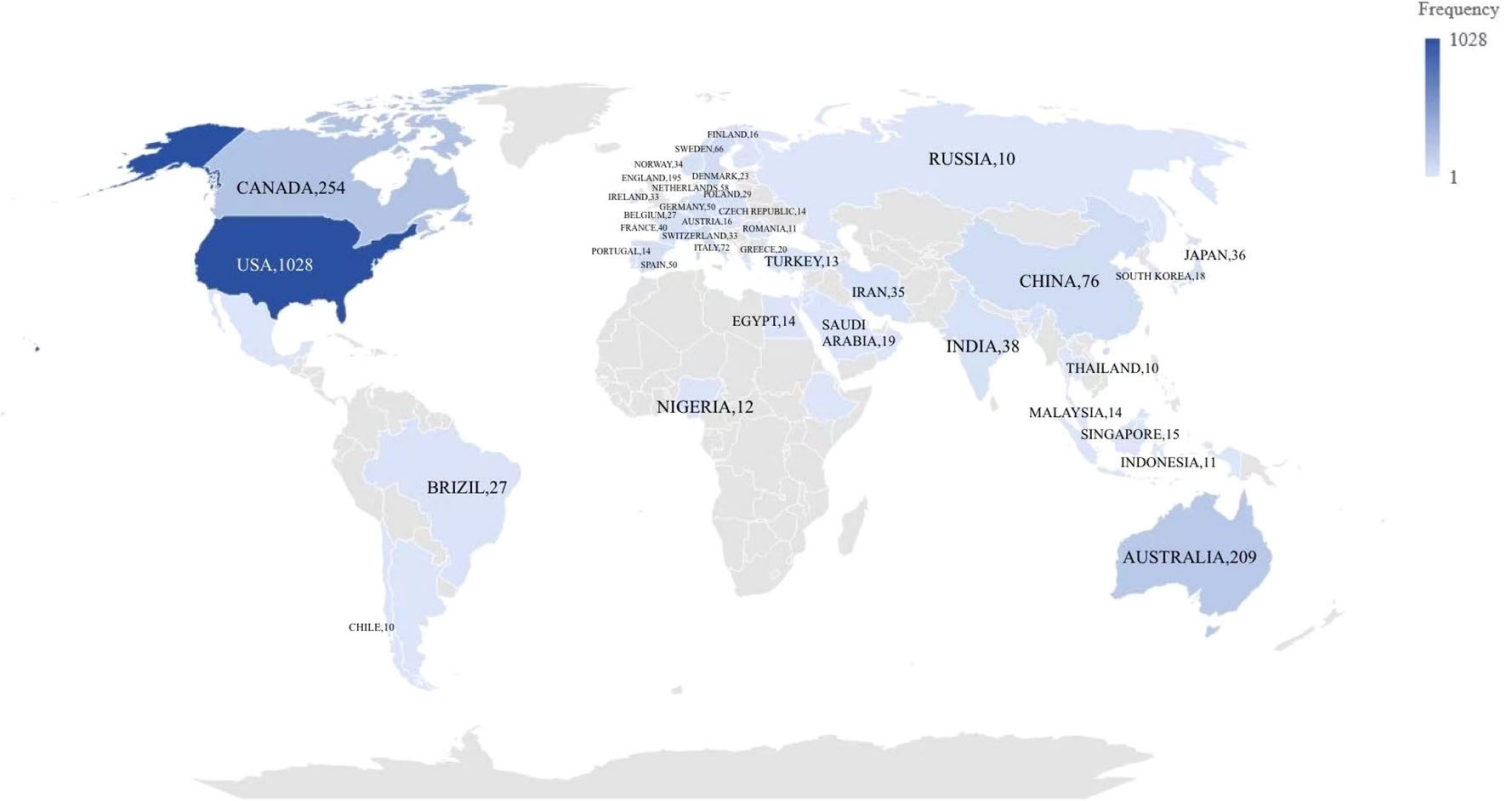
World map based on total number of publications in different countries/areas.

**Figure 4:**
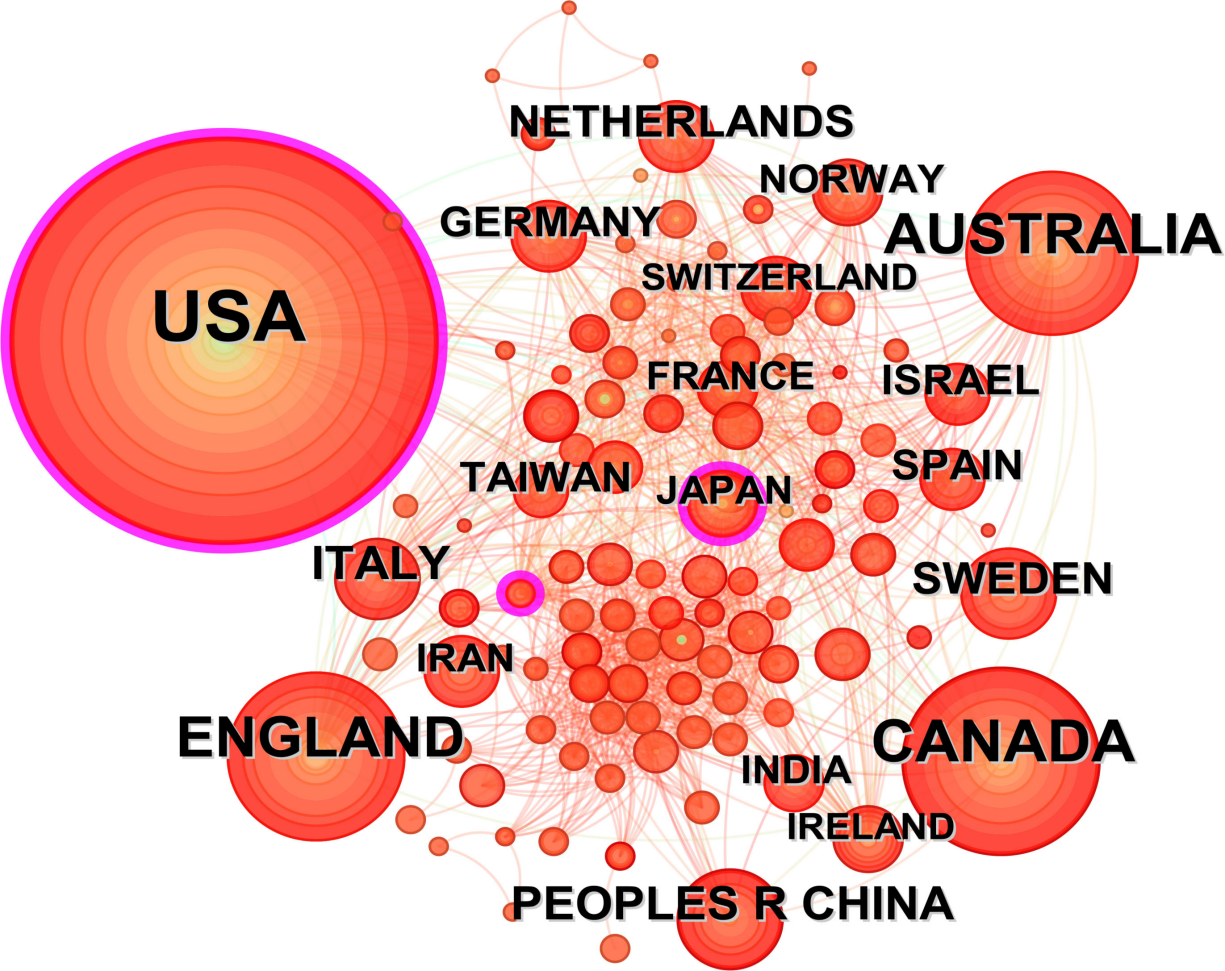
National/regional publications and cooperation networks.

**Figure 5:**
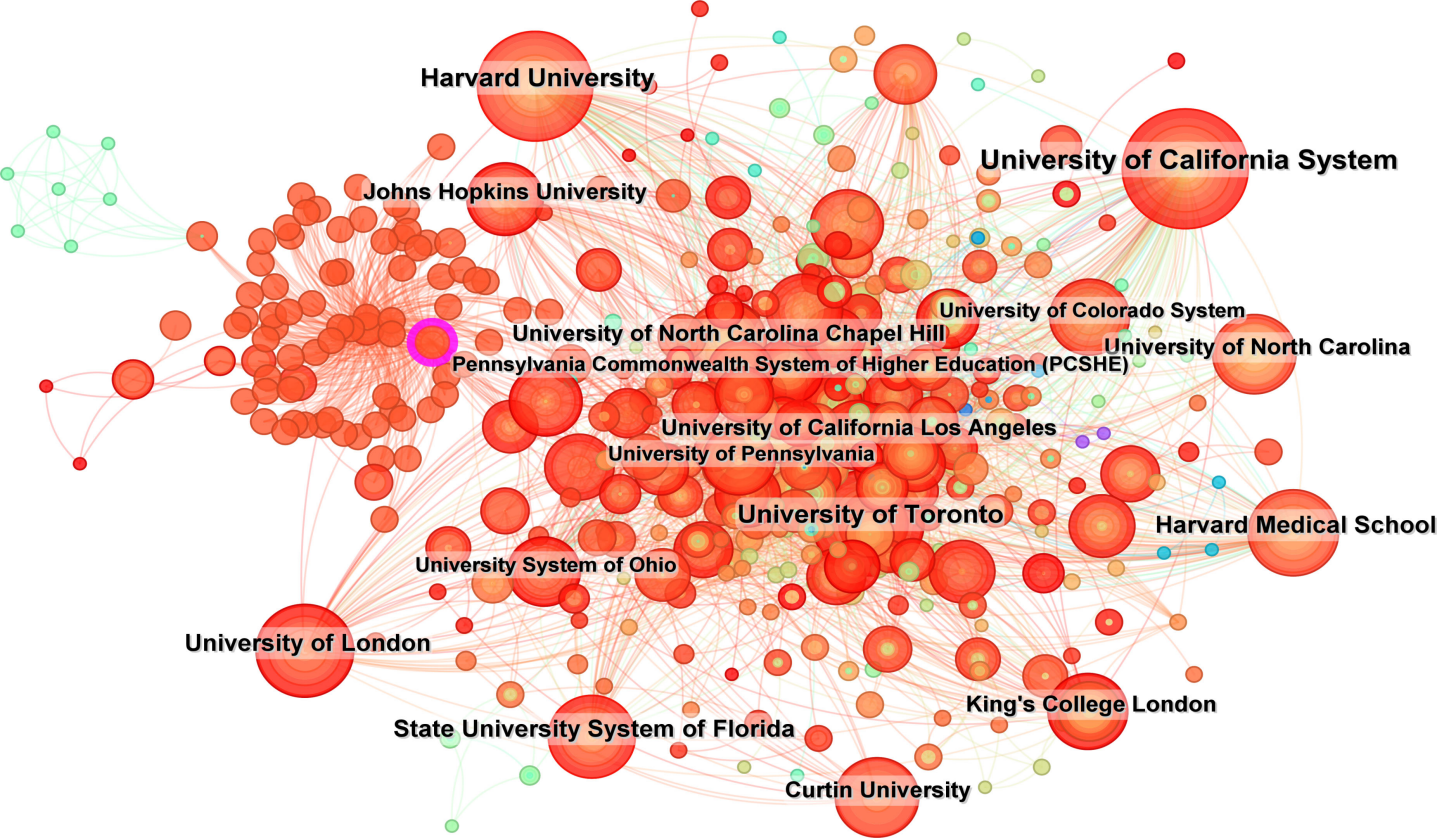
Institutional collaboration network diagram.

**Figures 3** and **4** illustrate the global dissemination of research outputs in this domain. The United States leads with 1,028 relevant studies, demonstrating both the earliest research initiation (centrality index = 0.12) and sustained dominance in the field. This is followed by Canada (254), Australia (209), the United Kingdom (195), and China (76). **Figure 5** and **Table 1** present the top 10 institutions by publication volume in autism family therapy research, with the University of California System ranking first at 132 publications. Harvard University (100), the University of Toronto (91), the State University System of Florida (79), and the University of London (73) follow closely. Notably, the majority of these top institutions are U.S.-based academic powerhouses, with only a small number from other national education systems. This underscores the dominance of American universities in autism family therapy research, attributed to their internationally leading research platforms and academic excellence. It should be noted that Harvard University and Harvard Medical School are listed separately in the dataset but are administratively affiliated within the same institutional network.

**Table 1:** Top 10 organizations publishing research on family therapy for autism.

Rank	Institution	Country	Count	Centrality
1	University of California System	USA	132	0.08
2	Harvard University	USA	100	0.09
3	University of Toronto	Canada	91	0.03
4	State University System of Florida	USA	79	0.03
5	Harvard Medical School	USA	74	0.07
6	University of London	England	73	0.05
7	University of North Carolina	USA	56	0.01
8	University of North Carolina Chapel Hill	USA	54	0.01
9	Curtin University	Australia	54	0.01
10	King’s College London	England	49	0.03

### 3.2 Authors

**Figure 6:**
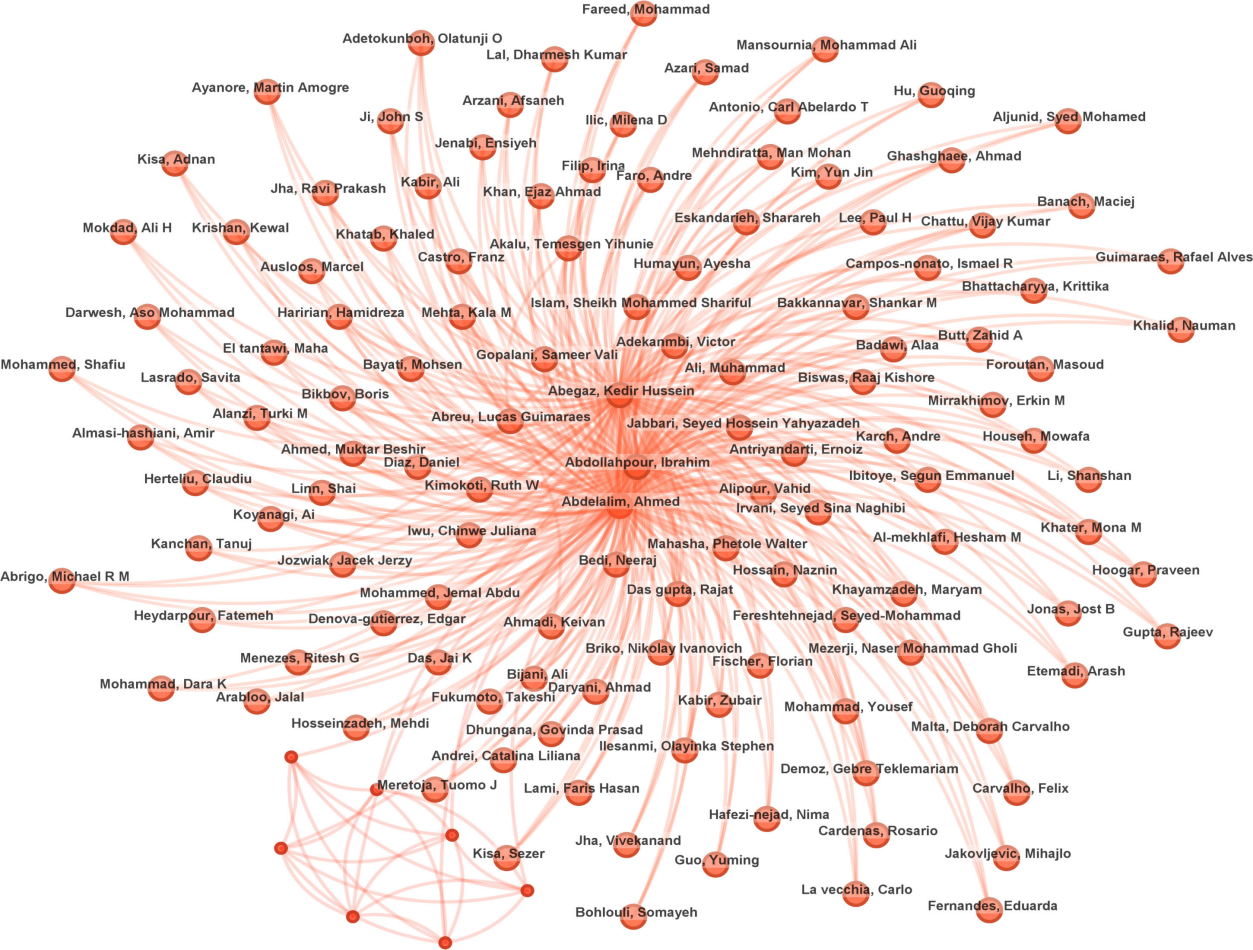
Author’s publications and collaborative networks.

A total of 873 authors contributed to the body of research on family therapy for autism (**Figure 6**). The network visualization uses nodes to represent individual researchers and edges to denote collaborative relationships, with node size proportional to publication output and edge thickness reflecting collaboration intensity. Color gradients from warm to cool tones encode temporal proximity, with warmer hues indicating more recent contributions. **Table 2** lists the top 10 most prolific authors, led by Baranek, Grace T. (19 publications), followed by Cordier, Reinie (13) and Storch, Eric A. (13). According to Price Law, the minimum number of articles by core authors (M) = 0.749 × √nmax (nmax is the number of articles by the most prolific authors), 15 M = 0.749 × 4.3 ≈ 3.22, rounded to an integer of 3, which means that the core authors have ≥4 publications. This identified 207 core authors responsible for 1,007 articles, accounting for 53.2% (1,007/1,891) of total publications—exceeding the 50% threshold for significant core contribution. The dense network structure observed in **Figure 6** indicates robust collaborative relationships among researchers in this field.

**Table 2:** Most important authors of family therapy for autism.

Rank	Frequency	Year	Author
1	19	2012	Baranek, Grace T
2	13	2017	Cordier, Reinie
3	13	2013	Storch, Eric A
4	10	2017	Vaz, Sharmila
5	10	2019	Weiss, Jonathan A
6	9	2017	Falkmer, Torbjorn
7	9	2014	Little, Lauren M
8	7	2019	Ghanouni, Parisa
9	6	2014	Watson, Linda R
10	6	2017	Anagnostou, Evdokia

### 3.3 Keyword clustering analysis

**Figure 7:**
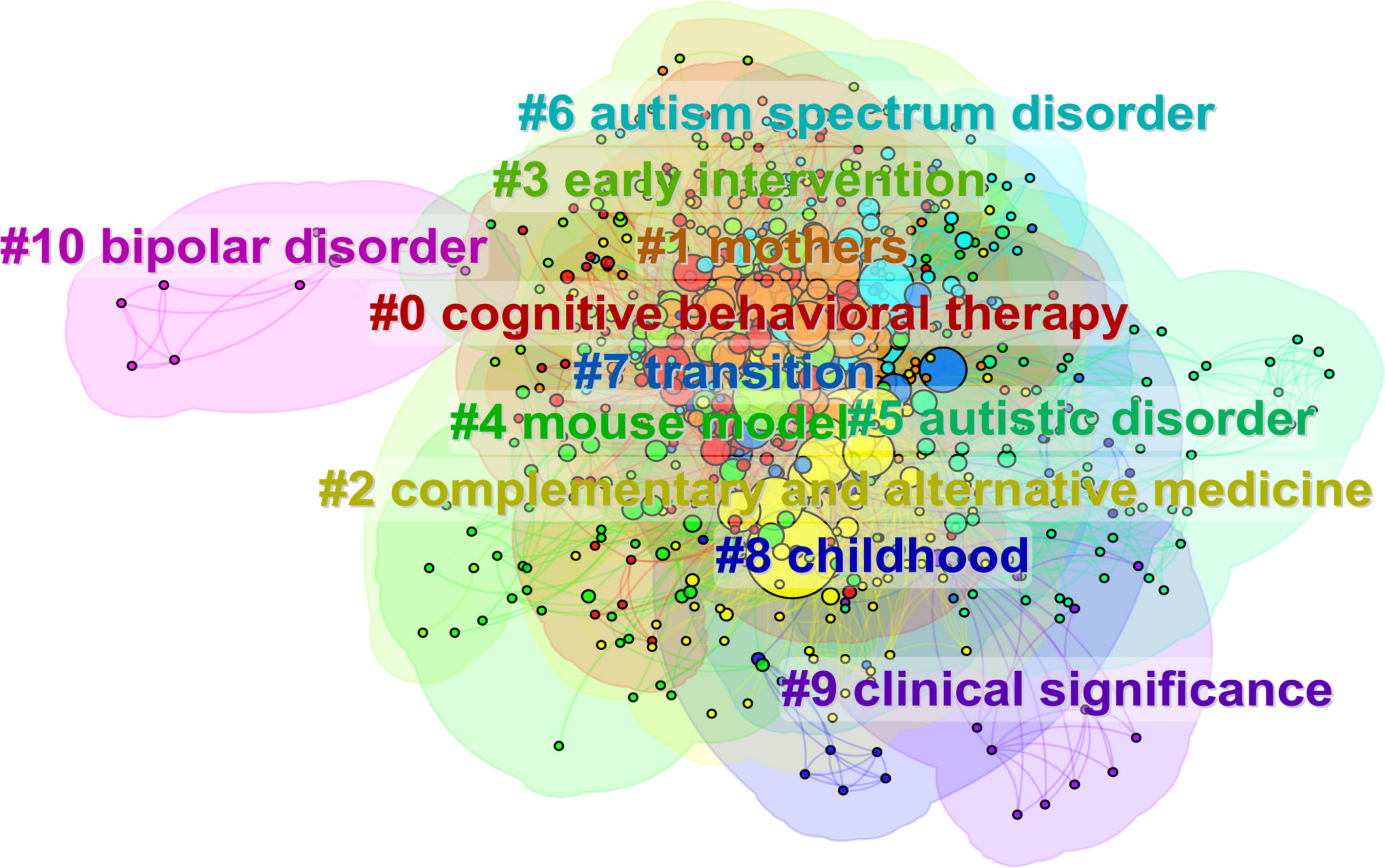
Visual keyword clustering analysis.

**Figure 7** displays the keyword co-occurrence network derived from the literature using the Log-Likelihood Ratio (LLR) algorithm, with clusters labeled #0 through #10. The visualization reveals 11 distinct thematic clusters, as visualized in **Figure 7**. The average silhouette value (*S* = 0.7833) exceeds the 0.5 threshold, indicating strong cluster validity and coherence. The compact distribution of clusters in the mapping suggests high inter-cluster relatedness and thematic convergence among research topics. **Table 3** presents weighted keywords within each cluster, highlighting their relative importance.

**Table 3:** Analysis of the top 10 keyword clusters in the research area of family therapy for autism.

Cluster ID	Size	Silhouette	Mean (year)	Label
0	108	0.562	2011	Cognitive–behavioral therapy; anxiety; obsessive–compulsive disorder; randomized controlled trial; psychotherapy
1	107	0.587	2014	Mothers; parenting stress; social support; acceptance and commitment therapy; family functioning
2	85	0.822	2006	Complementary and alternative medicine; autism spectrum disorders; double blind; melatonin; prevalence
3	81	0.652	2013	Early intervention; music therapy; parent coaching; joint attention; young children
4	67	0.76	2012	Mouse model; activation; epilepsy; schizophrenia; animal models
5	59	0.83	2002	Autistic disorder; haplotype block; mental retardation; association study; autism spectrum disorder
6	59	0.721	2015	Autism spectrum disorder; health disparities; occupational therapy; primary care; health services
7	34	0.799	2013	Transition; young adults; students; parent expectations; employment
8	108	0.562	2011	Cognitive–behavioral therapy; anxiety; obsessive–compulsive disorder; randomized controlled trial; psychotherapy

Integrating these cluster findings, early intervention emerges as a central research theme in autism family therapy. Subthemes include cognitive function training, attention deficit intervention, and family-based vocational rehabilitation, which collectively constitute the primary focus areas within this domain.

### 3.4 Keyword

**Figure 8:**
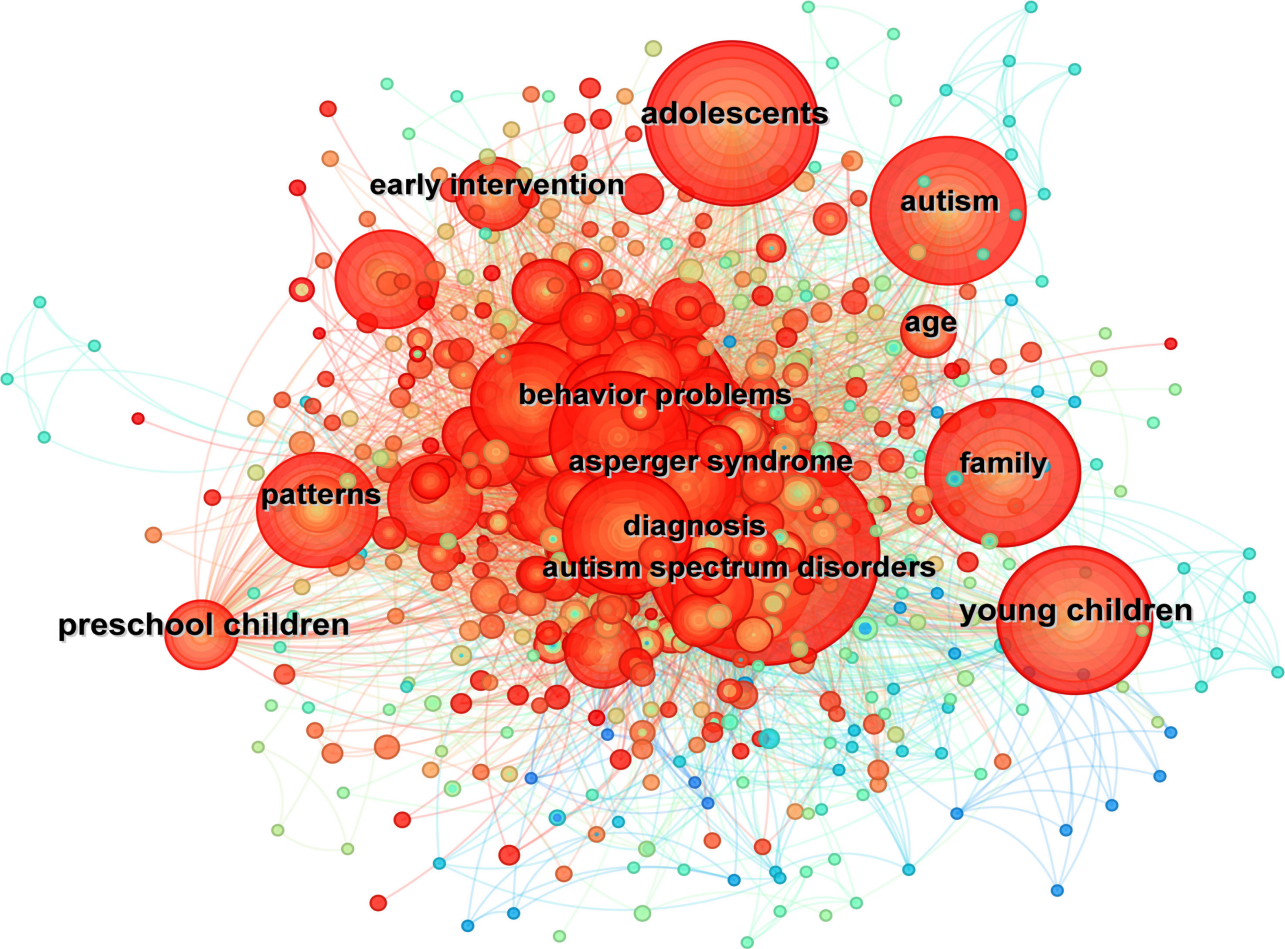
Network of keywords for family therapy for autism.

Keywords serve as highly condensed representations of scientific research features, reflecting disciplinary hotspots and inter-theme connections. Keywords were extracted using CiteSpace, which also constructed a visual mapping based on keyword co-occurrence relationships. In **Figure 8**, each node represents a keyword, with node size proportional to keyword frequency. Edges connect related keywords, with line thickness indicating co-occurrence strength. Collectively, the visualization depicts thematic associations and their relative prominence. Notably, **Figure 8** reveals two primary research trajectories. The first focuses on early intervention within home environments, including cognitive training, behavioral modification for children/adolescents with autism, and family-centered diagnostic practices. The second trajectory highlights emerging trends in quality of life enhancement for autistic families, communication skill development in children, and vocational rehabilitation programs for adult patients. These findings indicate an evolving research landscape balancing traditional clinical interventions with broader societal support systems.

### 3.5 Keyword surfacing chart

**Figure 9:**
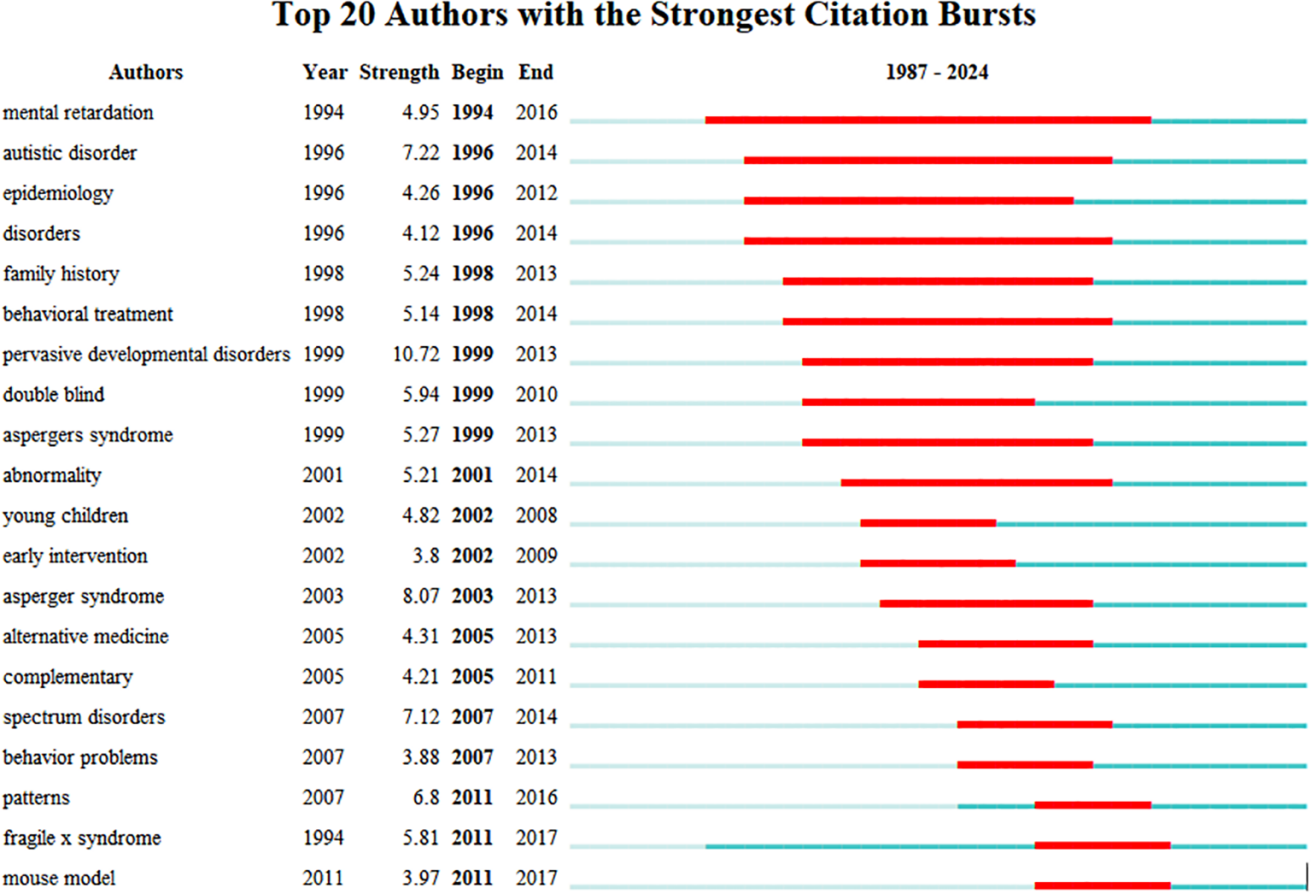
Top 15 most cited keywords.

Keyword emergence analysis focuses on algorithmically deriving research hotspots reflecting the emergence or continued attention over a period of time, reflecting the changes and trends of hotspots in the research field. The γ value was adjusted according to the co-occurrence of keywords, respectively, to obtain the keyword emergence analysis, with the blue line indicating the time interval and the red line indicating the time period in which the keyword emergence was found. “Children,” “autism spectrum disorder,” and “adolescents” are the earliest (1994) terms to appear. “Pervasive developmental disorder” is the earliest (1994) term to appear. “Pervasive developmental disorder” maintains a high intensity of occurrence from 1999 to 2013, with an intensity of 10.72, the highest intensity of emergence, suggesting that most children with autism are accompanied by developmental disorders, and that the US *Diagnostic and Statistical Manual of Mental Disorders Fourth Edition* (*DSM-IV*) includes autism as one of the disorders of pervasive developmental disorders. “Mental retardation” is the most frequently occurring keyword; most children with autism have varying degrees of mental retardation, and restoring the intelligence of children with autism has always been a central issue in rehabilitation. “Early intervention” and “patterns” are the most popular keywords in this field in the past 10 years, which indicates that the research on family therapy for autism is increasingly focusing on early intervention for children with autism at home as well as on intervention models (**Figure 9**).

### 3.6 Keyword timeline analysis

**Figure 10:**
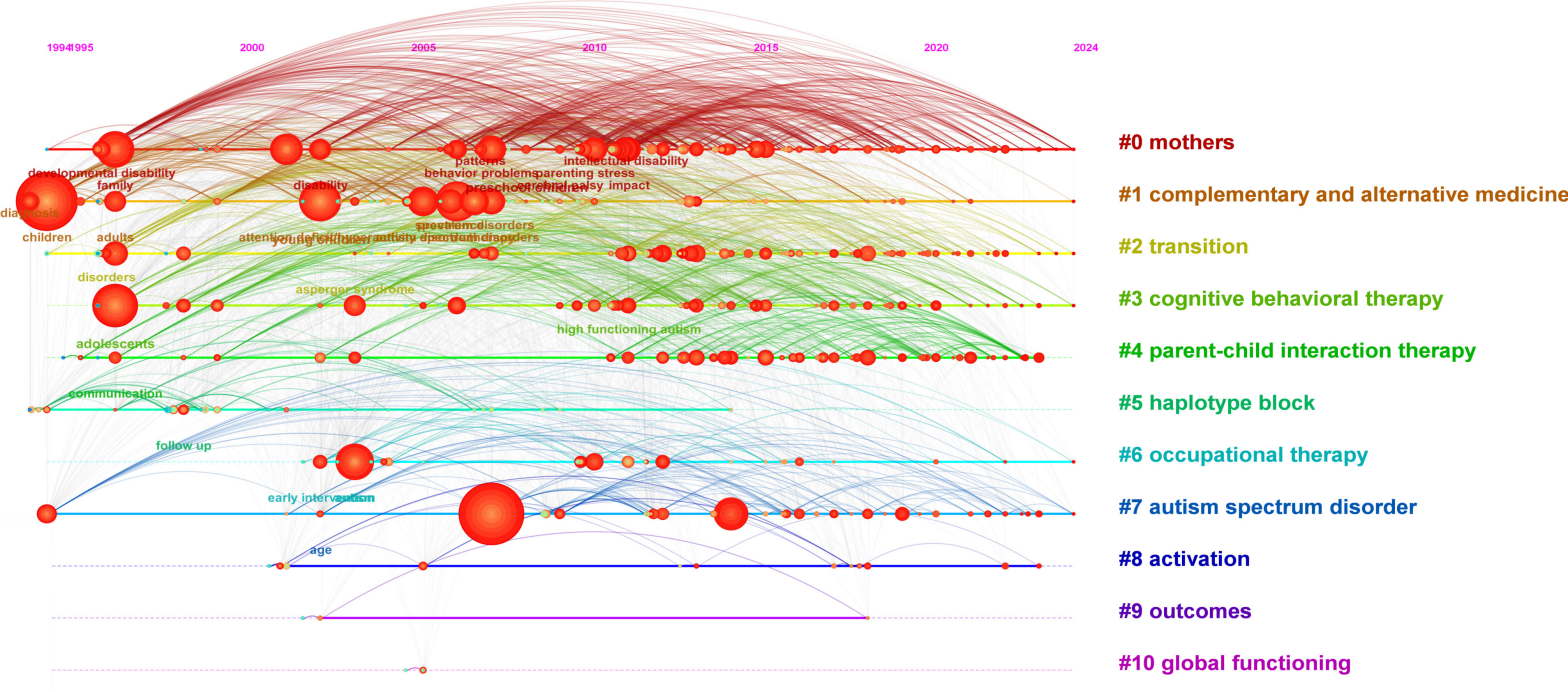
Keyword timeline chart.

In the keyword timeline view, the *x*-axis is the year in which the keyword appears in the cluster, and the *y*-axis is the cluster in which each keyword is located, which further shows the occurrence, end, and time trend of each cluster, which can reflect the importance of a specific cluster and the period of its distribution. According to the picture, it can be seen that from 1996 to 2010, autism research focused on children’s communication ability and early intervention; from 2010, the research hotspots gradually shifted to parent training, family therapy system, and parental mental health; from 2020 to 2024, the research hotspots shifted to the importance of family therapy for the autistic family intervention model, the emotional management ability of children with autism, comprehensive family wellbeing index, and caregiver psychology (see **Figure 10**). 

### 3.7 Journal double image overlay

**Figure 11:**
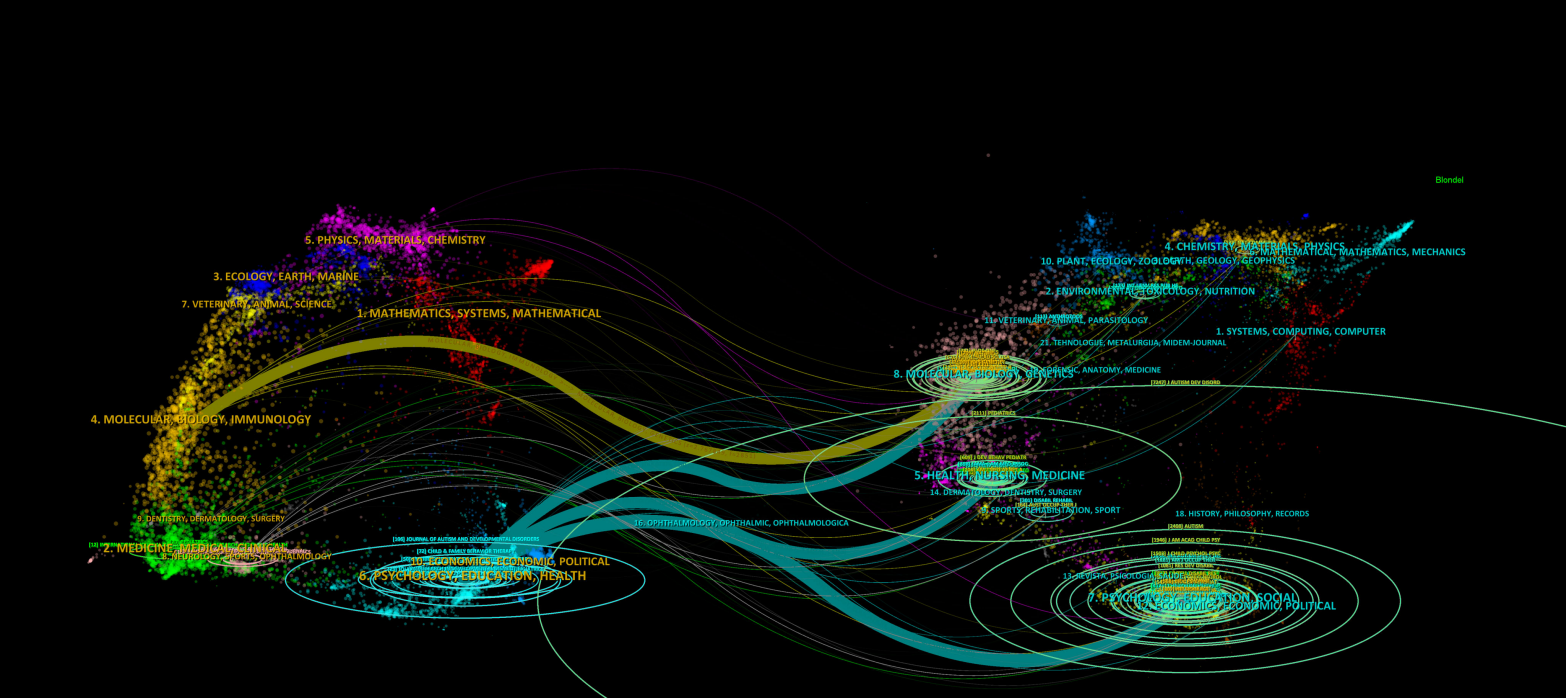
Journal double image overlay.

The superimposed journal overlay map (**Figure 11**) visualizes citation relationships between source and target journals, revealing interdisciplinary knowledge diffusion patterns in autism family therapy research. Cited journal clusters spanning economics, psychology, education, rehabilitation sciences, and genetics form the foundational knowledge base for understanding psycho-educational health dimensions in autism populations. For example, the *Journal of Autism and Developmental Disorders* (psychology), *Autism Research* (genetics), and *Rehabilitation Psychology* (rehabilitation sciences) serve as core knowledge hubs in this domain.

A newly emerged cluster linking Environmental Toxicology and Clinical Pharmacology highlights an evolving subfield focused on exploring autism’s environmental etiology and developing targeted pharmacotherapeutic interventions. This aligns with recent advancements examining interactions between genetic vulnerabilities, toxicant exposures, and pharmacological responses in autistic individuals.

The overlay map demonstrates robust translational research networks integrating three key dimensions: (1) psycho-educational models such as applied behavior analysis for skill development; (2) economic evaluations assessing cost-effectiveness of family-based interventions; and (3) pharmacogenetic studies investigating personalized medication responses. These integrations underscore the value of interdisciplinary collaboration in addressing autism’s multifactorial nature and improving rehabilitative outcomes.

### 3.8 Analysis of co-cited literature

The co-cited literature helps us to understand the research themes and development context of the field, and these co-cited literature forms the knowledge base of the study. After excluding some of the literature that is not in the field of this study, the 10 most cited of these are shown in **Table 4**. The most frequently cited articles were those published by Hayes SA, Pickles A, and Karst J, respectively. These articles focused on stress in families of children with ASD, parent-mediated social interventions for children with autism, and family intervention models for autism.

**Table 4:** Top 10 co-cited literature on family therapy for autism.

Rank	Author	Journals	DOI	Year	Citation
1	Hayes SA	*J AUTISM DEV DISORD*	10.1007/s10803-012-1604-y	2013	21
2	Pickles A	*LANCET*	10.1016/s0140-6736(16)31229-6	2016	18
3	Karst J S	*CLIN CHILD FAM PSYCH*	10.1007/s10567-012-0119-6	2012	17
4	Kinnear SH	*J AUTISM DEV DISORD*	10.1007/s10803-015-2637-9	2016	15
5	Bearss K	*JAMA-J AM MED ASSOC*	10.1001/jama.2015.3150	2015	15
6	Estes A	*AUTISM*	10.1177/1362361309105658	2009	14
7	Sutherland R	*INT J SPEECH-LANG PA*	10.1080/17549507.2018.1465123	2018	13
8	Lindgren S	*PEDIATRICS*	10.1542/peds.2015-2851O	2016	12
9	Nevill RE	*AUTISM*	10.1177/1362361316677838	2018	12
10	Dunn W	*AM J OCCUP THER*	0.5014/ajot.2012.004119	2012	12

## 4 Discussion

This paper utilizes CiteSpace software to visually and systematically analyze the literature on family interventions for autism. Through the generated knowledge graphs, it becomes evident that, since the beginning of the 21st century, an increasing number of researchers have recognized the significant role of family in improving the core symptoms of autism, with a particularly rapid growth rate observed since 2011. Several factors may contribute to this trend. First, the home environment serves as the initial and primary setting for children, offering a unique advantage over institutional rehabilitation. Second, parent-mediated training more closely mirrors real-life scenarios, enhancing its effectiveness.

Moreover, most studies on family interventions for autism have predominantly focused on regions such as North America and Europe, where research collaborations are more extensive. This suggests that these regions are at the forefront of family intervention research in autism. Beyond North America and Europe, China has published the third-highest number of articles (76); however, its centrality remains low (0.01). This indicates that despite a relatively high publication volume, China still lacks high-impact studies, possibly due to its later entry into this research field compared to Western countries.

By summarizing high-frequency keywords, performing cluster analysis, constructing a keyword clustering timeline, and conducting burst keyword analysis, key developmental trends in the field can be identified. At the end of the 20th century, research in this domain primarily focused on the relationship between genetic factors and ASD onset. Entering the 21st century, studies began emphasizing early family-based interventions and cognitive–behavioral therapy for children with autism. As research progressed, interventions increasingly targeted caregivers, aiming to enhance social interaction of children with autism. In later stages, caregiver-based interventions expanded their focus to areas such as social engagement, attention, emotional regulation, and pragmatic language skills.

In recent years, a growing body of literature has shifted its focus to the broader impact of autism on family functioning, particularly concerning financial burden and parental stress. Analyzing authors’ publication networks and cited journals can enhance our understanding of the relationship between family and autism, thereby enriching the theoretical foundation of the field and improving the quality of related research. Mapping authors’ collaborative networks provides insights into key scholars in family intervention research for autism. Among them, the most prolific researcher is Grace T. Baranek, whose primary research interests include neurodevelopment and intervention strategies for children with autism. Baranek’s recent publications focus on parent–child relationships in autism and early family-based interventions. From a broader perspective, the collaboration network reveals that Ahmed Abdelalim and Kefir Abegaz work closely together. However, their nodes are concentrated in the central region of the network, indicating a relatively fragmented research landscape. Strengthening interdisciplinary and cross-institutional collaborations could help advance the field further.

Examining high-frequency co-cited literature provides a quick overview of foundational studies in the field. The most frequently co-cited paper is a meta-analysis by Stephanie A. Hayes and Watson (23), which found that families of children with autism experience significantly high levels of stress, with a large effect size. Another highly co-cited study by Andrew Pickles et al. (24) conducted a randomized controlled trial (RCT) on a parent-mediated social intervention. Their findings demonstrated that the Preschool Autism Communication Trial (PACT) had long-term positive effects on children with autism.

### 4.1 Effectiveness and impact of autism family interventions

Since the introduction of family interventions for autism, substantial evidence has demonstrated their positive effects not only on the symptoms of children with autism but also on various other aspects of family wellbeing. Research has shown that parenting a child with autism imposes significant stress on parents, negatively affecting their mental health (25) and potentially influencing the development of young children with autism (26).

In the absence of specialist knowledge, group-based parent training has been identified as a cost-effective approach for providing education and support to parents. This method has proven effective for children with intellectual and physical disabilities (27). Family-Focused Psychoeducational Therapy (FFPT) is another intervention designed to reduce family stress and negative emotional responses, enhance psychosocial functioning, and strengthen social and family support systems during the child’s developmental process (28). A study by Zhou et al. (29) applied FFPT to families of children with autism, demonstrating that FFPT improved parental self-efficacy and psychological wellbeing, enhanced communication skills, and reduced maladaptive emotional responses.

Additionally, research suggests that family interventions can improve parent–child relationships. Parents who actively engage in their child’s therapy are better able to interpret and respond to their child’s communicative expressions (30). Increased parental involvement in autism interventions is associated with reduced parenting challenges and improved family dynamics. However, there is a lack of large-scale RCTs to fully validate these findings. Studies have also suggested that parent-mediated interventions delivered via telemedicine provide a cost-effective alternative to traditional in-person therapy (31, 32).

### 4.2 Main forms of family interventions

#### 4.2.1 Parent-mediated interventions

Parent-mediated interventions empower parents to take on the role of therapists, implementing intervention strategies directly with their children (33). Evidence indicates that parent training in intervention techniques yields significant benefits for children with autism, increasing parental knowledge and confidence in managing their child’s condition (34). In addition to improving child outcomes, parent training also provides psychosocial support for parents, leading to reductions in parental mental health issues, increased understanding of their child’s challenges, improved parent–child interactions, and enhanced social and communication skills in children with autism.

Parent-mediated interventions typically involve three key steps: (1) identifying potential intervention strategies, (2) training parents to implement these strategies, and (3) fostering collaboration between parents and therapists, with therapists providing ongoing feedback (35). However, research on parental self-efficacy in interventions remains limited, as parental self-efficacy is often not a direct outcome measure of these programs. Given that early reinforcement-based and behavioral interventions are the most commonly implemented strategies for children with autism, studies on parental self-efficacy often rely on indirect measures, such as parental wellbeing following family-based therapy. This methodological limitation contributes to the scarcity of research in this area.

#### 4.2.2 Telehome healthcare

Telehome healthcare is an emerging medical technology that utilizes digital platforms to deliver healthcare services over long distances (36). Compared to traditional face-to-face methods, telemedicine offers significant advantages in the early detection and diagnosis of ASD. Digital medical devices help address the increasing demand for early ASD screening and diagnosis while enhancing the standardization of ASD assessment and management (37).

Currently, telehome healthcare can be categorized into two main forms: caregiver-mediated home healthcare and therapist-mediated home video interventions.

One widely studied caregiver-mediated intervention is Improving Parents as Communication Teachers (Project ImPACT Online), an evidence-based parent training program for children with ASD developed by Professors Ingersoll and Dvortcsak at Michigan State University. This intervention integrates self-directed, internet-based telemedicine with therapist guidance to train parents in promoting core developmental skills, including social engagement, communication, imitation, and play, within naturalistic settings (38, 39).

A modified version, Project ImPACT for Toddlers (PIT), was developed by Stahmer et al. (40) to target younger children. In this model, physicians deliver the intervention program through parent-mediated behavioral strategies. Findings indicated significant improvements in parent–child interactions, as well as substantial gains in children’s social and communication skills. Additionally, Hao et al. (2009) expanded on the ImPACT framework with the Skills and Knowledge of Intervening project, designed to enhance language and communication abilities in children with ASD (38).

Another key parent-mediated telehealth intervention is the PACT, a communication-focused intervention based on Naturalistic Developmental Behavioral Intervention (NDBI) principles. Recent adaptations of PACT to telemedicine include Pediatric Autism Communication Therapy-Generalized (PACT-G) and PACT-plus, delivered via tele-video conferencing (41).

Therapist-mediated telehome healthcare involves real-time online interactions between therapists and children, with caregivers required to be present. Research has demonstrated the feasibility and effectiveness of this approach, with high levels of parental adherence, engagement, and satisfaction (42). In a study by Marino et al. (43), children with ASD received therapist-guided telemedicine training for 4 months. Results showed that online therapy was comparable to in-person rehabilitation in terms of language skill improvements. However, while online interventions were effective in improving pragmatic language skills, children who participated in offline rehabilitation exhibited greater overall enhancements in socio-pragmatic abilities.

Family-based telemedicine interventions not only facilitate therapy delivery but also enhance parental skills training, ultimately benefiting both children with ASD and their families.

### 4.3 Main intervention techniques for family interventions

#### 4.3.1 Applied behavior analysis

Applied behavior analysis (ABA) is based on the behaviorist theory that both simple and complex behaviors can be taught through reinforcement and consequences. This approach aims to enhance behavioral, cognitive, and social communication skills in children with ASD (44). ABA was among the first methods implemented in family interventions for children with ASD. Research suggests that parents trained in behavioral therapy techniques can successfully apply ABA strategies in home settings (45).

A study by Marino et al. (46) randomly assigned 42 parents of children with ASD to receive 12 sessions of ABA-based interventions, delivered either individually or in small groups, with and without remote assistance. Results indicated that remotely assisted ABA interventions significantly improved parental knowledge and child behavior management while also reducing parental stress related to child behavior.

#### 4.3.2 Early Start Denver Model

The ESDM is an NDBI designed to meet the needs of very young children with ASD (47). ESDM emphasizes parent involvement, requiring caregivers to undergo training and collaborate with a primary therapist. Parents implement ESDM strategies in daily activities and help guide treatment goals. Studies suggest that parental use of technology enhances the effectiveness of ESDM interventions (48, 49).Compared to previous ASD interventions, ESDM has demonstrated greater benefits in cognition, adaptability, language development, motor function, and daily living skills in young children (50). Research indicates that families engaging in ESDM for at least 5 h per week acquire proficient skills in its application (51). Parents and family members play a crucial role in the success of ESDM programs. Rogers et al. (52) found that a 12-week, low-intensity (1 h of therapist contact per week) parent-mediated ESDM intervention for children aged 14–24 months significantly improved child communication skills, enhanced parent–child interactions, and reduced parental stress. These findings highlight not only the direct benefits of ESDM for children but also its positive effects on the parent–child relationship.

ESDM also influences neural activity associated with social cognition. Dawson et al. (47) investigated whether ESDM interventions impact electroencephalographic (EEG) activity by randomly assigning 48 children with ASD (aged 18–30 months) to either an ESDM group or a community-based intervention group. At the end of the intervention, EEG results showed that children in the ESDM group, along with neurotypical peers, exhibited shorter Nc latencies and higher cortical activation (characterized by decreased alpha waves and increased theta waves) during face-viewing tasks. In contrast, children with ASD in the community intervention group displayed the opposite pattern.

Further research by Aaronson et al. (53) examined the relationship between ESDM and Mu rhythms. Their study randomly assigned 48 children with ASD (aged 18–30 months) to either an ESDM or a community intervention group. Children in the ESDM group exhibited significantly greater Mu rhythm attenuation when observing their parents or caregivers perform grasping movements, compared to unfamiliar individuals performing the same action. This suggests that ESDM has a potential impact on neural mechanisms underlying social cognitive processes.

As a family-centered intervention, ESDM holds significant promise for the future. Its family-based approach enables parents to integrate skill training into daily routines, making treatment more accessible and reducing family burden while improving overall family wellbeing.

#### 4.3.3 Pivotal response training

Pivotal response training (PRT) is based on ABA principles but focuses on targeting pivotal developmental areas to promote the generalized acquisition of other non-targeted skills. By intervening in critical skills, PRT aims to enhance overall learning, social interaction, and behavioral outcomes in children with ASD (54).

Drapalik et al. implemented an online PRT model, consisting of eight 20-min training sessions, to reduce ASD-related symptoms. The results indicated increased parental confidence in implementing PRT strategies. Another study by Antonio et al. demonstrated that a 12-week parent-implemented PRT program enhanced both parental and child functional communication skills. Minjarez et al. (55) suggested that PRT instruction in group settings could expand access to in-home services at a lower cost. However, to date, no RCTs have been conducted on group-based PRT interventions. Future research should explore the feasibility and efficacy of PRT delivered in a panel format.

#### 4.3.4 TEACCH

The Treatment and Education of Autistic and Communication-Handicapped Children (TEACCH) program is a structured, evidence-based training model designed for individuals with ASD. It has been widely recognized by educators and therapists worldwide (56). A study by Turner-Brown et al. (57) found that families engaged in TEACCH interventions for 6 months experienced significant reductions in stress and improvements in overall wellbeing. Although no direct treatment effects were observed for core ASD symptoms, significant gains were reported in social communication skills. Additionally, a review of autism treatments concluded that TEACCH meets the criteria for an evidence-based, comprehensive intervention model for children with ASD (58) (**Table 5**).

**Table 5:** Main intervention techniques in family therapy.

Intervention	Key Principles	Outcomes
Applied behavior analysis	Reinforcement-based strategies	Improves behavioral management and reduces parental stress
Early Start Denver Model	Naturalistic developmental–behavioral focus	Enhances cognition, language, and social skills
Pivotal response training	Targets pivotal developmental areas	Promotes generalized skill acquisition
TEACCH	Structured environment and visual supports	Reduces parental stress and improves social communication

### 4.4 Multidisciplinary research on family interventions

A wide range of family interventions for ASD are currently available, and therapists should tailor their selection to address the unique symptoms of each child. A review of the existing ASD literature suggests that family interventions hold significant promise for the future. Family-based interventions have demonstrated positive effects on children, including improvements in core symptoms. However, they are also associated with increased parental stress and varying impacts on family wellbeing, as well as reduced financial burden. Despite these advantages, aspects such as attention, eye gaze, and learning ability in children with ASD have not been extensively validated in the literature. Thus, further research is needed, and multidisciplinary collaboration is essential to maximize the benefits of family-based interventions.

Artificial intelligence (AI) encompasses core technologies such as data discovery and learning, human–computer interaction, and knowledge and data intelligence processing (59). AI has shown potential in improving core ASD symptoms, but the vast range of available technologies has led to a lack of standardized treatment protocols. Linda et al. employed a computer-based social skills intervention, FaceSayTM, with children with ASD, demonstrating improvements in eye gaze, joint attention, and facial recognition skills (60). However, whether such technologies can be effectively implemented in home settings remains unclear. This highlights AI’s potential in ASD interventions and suggests that future research should focus on designing AI-based interventions specifically for home use, addressing both core ASD symptoms and broader aspects of family functioning.

### 4.5 Summary

Currently, family intervention approaches primarily involve either direct or indirect therapist-guided interventions delivered through online video conferencing or telephone consultations. Future research could explore software-based interventions that provide structured guidance for caregivers through interactive tasks. While most studies have focused on improving core ASD symptoms, limited research has examined the broader impact of family interventions on overall family functioning.

Caregiver stress plays a pivotal role in the developmental outcomes of children with ASD, as parental mental health is closely linked to child progress. Family interventions have been shown to positively influence children’s verbal abilities, reduce repetitive and stereotyped behaviors, and improve daily living skills. However, in terms of social interaction, family-based interventions face limitations, as they often lack opportunities for children to engage with peers of the same age. This may result in slightly poorer social skills compared to children receiving institution-based rehabilitation. Future research should explore strategies to simulate peer social interactions within family-based intervention models.

Over the past three decades, substantial progress has been made in family-based autism interventions, supported by a growing body of high-quality research. The use of visual analytics can help identify general trends and research hotspots in this field. Moving forward, key areas of research will likely include the effects of family-based early interventions on the social competence of children with ASD, parental stress, associated psychological challenges, and the development of innovative family intervention models.

### 4.6 Cultural and methodological limitations

Most studies on family interventions originate from Western countries, such as the United States and Canada, where individualistic cultural values may shape intervention approaches differently from those in collectivist societies, such as China. In Western settings, parent-mediated interventions often emphasize child-centered goals, whereas Asian families may prioritize family harmony and parental authority. Future research should investigate how cultural factors influence intervention effectiveness and develop culturally adaptive intervention models.

Additionally, many of the included studies lack rigorous methodological designs, limiting the generalizability of their findings. Future research should prioritize well-controlled experimental studies, including RCTs, to ensure robust and reproducible results.

This study is subject to a number of limitations. Firstly, only relevant literature from the WOS core dataset was included in this study for inclusion in the analyses, which may produce some bias in the results. Second, although CiteSpace is a professional bibliometric tool that provides objective analyses, there may be some subjective bias as different researchers may have different views and interpretations of the same content. Finally, the quality of the literature varies and global results on the overall structure of the research field may be more limited.
